# Evaluation of Properties and Micro-Characteristics of Waste Polyurethane/Styrene-Butadiene-Styrene Composite Modified Asphalt

**DOI:** 10.3390/polym13142249

**Published:** 2021-07-09

**Authors:** Bei Chen, Fuqiang Dong, Xin Yu, Changjiang Zheng

**Affiliations:** College of Civil and Transportation Engineering, Hohai University, Nanjing 210098, China; chenbhhu@126.com (B.C.); hhu_yuxin@126.com (X.Y.); zhenghhu@sina.com (C.Z.)

**Keywords:** asphalt pavement, waste polyurethane, modified asphalt, dynamic rheology, micro-characteristics

## Abstract

In order to solve the problems caused by asphalt diseases and prolong the life cycle of asphalt pavement, many studies on the properties of modified asphalt have been conducted, especially polyurethane (PU) modified asphalt. This study is to replace part of the styrene-butadiene-styrene (SBS) modifier with waste polyurethane (WP), for preparing WP/SBS composite modified asphalt, as well as exploring its properties and microstructure. On this basis, this paper studied the basic performance of WP/SBS composite modified asphalt with a conventional performance test, to analyze the high- and low-temperature rheological properties, permanent deformation resistance and storage stability of WP/SBS composite modified asphalt by dynamic shear rheometer (DSR) and bending beam rheometer (BBR) tests. The microstructure of WP/SBS composite modified asphalt was also observed by fluorescence microscope (FM) and Fourier transform infrared spectroscopy (FTIR), as well as the reaction between WP and asphalt. According to the results of this study, WP can replace SBS as a modifier to prepare WP/SBS composite modified asphalt with good low-temperature resistance, whose high-temperature performance will be lower than that of SBS modified asphalt. After comprehensive consideration, 4% SBS content and 15% WPU content (4 S/15 W) are determined as the suitable types of WPU/SBS composite modified asphalt.

## 1. Introduction

As traffic load, climate and environment change, asphalt pavement disease has progressively worsened in recent times, with problems including rutting, cracking, spalling, etc. [1,2], reducing the life cycle of asphalt pavement, and increasing its maintenance cost, with a significant impact on the sustainable development of highways [3]. As the binder of pavement mixture, the traditional asphalt, has been unable to meet the demands of engineering design due to the limitation of its own chemical properties [4]. In order to solve the above diseases effectively, research on modified asphalt is of great significance in improving the performance and prolonging the life cycle of asphalt pavement.

Polymer modified asphalt technology was developed in the 1840s. In the 1930s, neoprene latex was used as the first modifier in the application of asphalt pavement engineering in Europe. By the 1950s, modified asphalt had been studied and applied in Asia and the United States [5]. In the research on asphalt modification, SBS modified asphalt has been studied most, mainly because adding SBS can effectively improve the high-temperature rutting resistance and low-temperature cracking resistance of asphalt, and effectively prolong the life cycle of asphalt pavement [6]. However, the production and application of SBS modified asphalt still have problems, including complex production technology, high price, and so on. Therefore, it is extremely urgent to find a low-cost and stable polymer to replace SBS modifier.

PU, as a polymer compound, is widely used in various industries for its excellent heat preservation and processability, accounting for 5% of the total plastic production in the world. With the continuous increase of PU production, PU waste is increasing at an annual rate of 20% for synthetic rubber, construction, coatings, electronic and electrical appliances, and automobile industries [7]. Currently, methods of dealing with WPU include: (1) burying and burning, traditional treatment methods that can cause great harm to water, air and soil; (2) a method by which WPU is crushed mechanically, before being used as filler, foam plastic, artificial soil, etc., —this is ineffective, with little economic value [8]. Therefore, WPU can be used as a new type of asphalt modifier. After adding WPU to asphalt, the durability and elastic recovery rate can be improved, and the plastic deformation resistance of asphalt under environmental load and other factors can be enhanced at the same time, so as to improve the performance of modified asphalt and realize recycling of WPU. The study has far-reaching significance in reducing the cost of materials and realizing the concept of green environmental protection.

At present, some researchers have studied the application of PU in asphalt, but studies on WPU in asphalt are still rare [9]. Among them, He et al. [10] optimized the preparation of PU modified asphalt, and discussed the influence of PU content on the properties of PU modified asphalt, indicating that the performance of modified asphalt can be improved with the increase of PU content, but that the content should not exceed 25%. Referring to Sun et al. [2], the performance and production cost of PU modified asphalt, base asphalt and SBS modified asphalt were compared, with the results showing that PU modified asphalt has better high-temperature performance and water stability but low-temperature performance which is lower than SBS modified asphalt. In the study of Yu et al. [11], a graphene oxide/PU composite modified asphalt was proposed, showing that composite modified asphalt can improve high-temperature performance of asphalt, elastic modulus and mild yield of asphalt, the mechanism of which has been explained from micromechanics. Therefore, it is feasible to seek a kind of economical and stable composite PU modified asphalt [12].

So far, there have been a lot of studies on the properties of finished PU asphalt, but no comprehensive and systematic evaluation on WPU modified asphalt. In this study, a kind of WPU/SBS composite modified asphalt with WPU and SBS modifier was prepared, and the preparation of WPU/SBS composite modified asphalt was explored. The basic performance of composite modified asphalt was analyzed in a base performance test and rotational viscosity test; moreover, high- and low-temperature performance and storage stability of WPU/SBS composite modified asphalt were tested in the dynamic shear rheometer (DSR) and bending beam rheometer (BBR) tests, to determine the performance grade (PG) of WPU/SBS composite modified asphalt. The resistance to permanent deformation of WPU/SBS composite modified asphalt was evaluated in the multiple stress creep recovery (MSCR) test. The microstructure changes of WPU/SBS composite modified asphalt were investigated by a fluorescence microscope (FM) test and Fourier transform infrared spectroscopy (FTIR) test.

## 2. Materials and Methods

### 2.1. Materials

#### 2.1.1. Asphalt

WPU/SBS composite modified asphalt samples were prepared from SK-based asphalt provided by a Korean asphalt plant (Seoul) with the penetration of 60/80, and its properties are shown in Table 1 and Table 2.

#### 2.1.2. Waste Polyurethane

WPU materials were obtained by crushing and processing waste plastic foam, with the particle size of WPU powder less than 0.075 mm. WPU is a kind of soft foam, which comes from aromatic compounds. Properties of WPU are shown in Table 3.

#### 2.1.3. Styrene-Butadiene-Styrene (SBS)

The styrene/butadiene ratio of SBS was 32/68. The structure of SBS is linear. Basic properties are presented in Table 4.

### 2.2. Preparation of Modified Asphalt

#### 2.2.1. Preparation of SBS Modified Asphalt

SBS modified asphalt was prepared by a FM300-DIGITAL shearing machine (Motlong, Wenzhou, China). First, 3 portions of base asphalt were taken, then melted at 175 °C. Adding 3%, 4% and 5% SBS modifier, base asphalt was sheared with a shearing machine at 3000 r/min for 1 h, heated to 180 °C, and then stirred with a mixer for 1 h.

#### 2.2.2. Preparation of WPU/SBS Composite Modified Asphalt

WPU/SBS composite modified asphalt was prepared by the process. First, 4 portions of base asphalt were taken, then melted at 175 °C. After adding 4% SBS modifier, base asphalt was sheared with a shear mixer at 3000 r/min for 1 h, then heated to 180 °C, with 5%, 10%, 15% and 20% of WPU, respectively, which was first sheared at 3000 r/min for 1 h, and then stirred with a mixer for 1 h.

According to the different content of SBS modifier in the study, SBS modified asphalt prepared with 3%, 4% and 5% SBS modifier was marked as 3 S, 4 S and 5 S, respectively. In the performance study, WPU/SBS composite modified asphalt prepared with 5%, 10%, 15% and 20% WPU was marked as 4 S/5 W, 4 S/10 W, 4 S/15 W and 4 S/20 W, respectively.

### 2.3. Specimen Preparation

**Figure 1:**
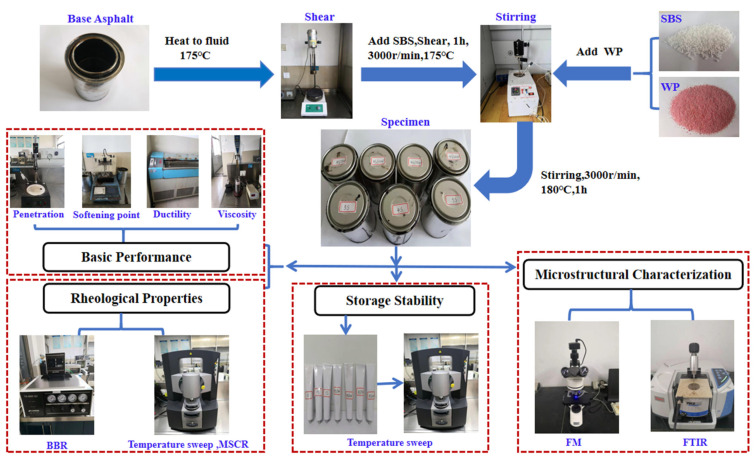
Flowchart of experiment planning of WPU/SBS composite modified asphalt.

According to AASHTO and ASTM experimental standards, the performance test and microstructure observation of WPU/SBS composite modified asphalt were carried out. Subsequently, basic properties, high-temperature and low-temperature rheological properties, storage stability and microstructure of WPU/SBS composite modified asphalt were evaluated by base performance test, rotational viscosity test, temperature scanning test, frequency scanning test, BBR test, segregation experiment, FM test and FTIR test, as shown in Figure 1.

### 2.4. Test Procedures

#### 2.4.1. Basic Performance Tests

(1) Basic performance tests of modified asphalt

In order to explore basic properties of WPU/SBS composite modified asphalt, penetration test, softening point test and ductility test were carried out at 5 °C according to technical requirements of ASTM and AASHTO polymer modified asphalt [17].

The temperature sensitivity of asphalt can be characterized by the penetration index (PI). The higher the PI index, the lower the sensitivity. Asphalt has a gel structure when PI is higher than or equal to 2, and a sol-gel structure when PI index is less than 2. The PI index can be deduced according to the following Formula (1) [12]:

(1) $$PI=\frac{1952-500\log(P25)-20Sp}{50\log(P25)-Sp-120}$$

where, *S*p is the softening point and *P*25 is the penetration at 25 °C.

(2) Rotational viscosity tests of modified asphalt

The viscosity of modified asphalt is one of important indexes of modified asphalt. The NDJ-1C Brinell rotational viscometer and No. 27 rotor (Changji, Shanghai, China) were used. At 135 °C, 145 °C, 155 °C, 165 °C and 175 °C, the viscosity of WPU/SBS composite modified asphalt was tested by rotational viscosity test.

#### 2.4.2. Rheological Property Tests

(1) Temperature Sweep Tests

According to the requirements of ASTMD 7175, a TA-AR1500EX dynamic shear rheometer (DSR) (25 mm diameter parallel plates, 1 mm gap) (Through asphalt sand, Tongsha, China) was used to scan at 46 °C, 52 °C, 58 °C, 64 °C, 70 °C, 76 °C and 82 °C. The storage modulus (G’), loss modulus (G”), phase angle and anti-rutting factor G^*^/sin *δ* of WPU/SBS composite modified asphalt were measured, to explain rheological properties of WPU/SBS composite modified asphalt at high temperature.

(2) Multiple Stress Creep Recovery (MSCR) Tests

According to AASHTO technical standard TP 70-10 (AASHTO 2013) [18], the ability to delay elastic deformation and recover WPU/SBS composite modified asphalt was evaluated to explore the resistance to permanent deformation of WPU/SBS composite modified asphalt, with the TA-AR1500EX DSR instrument in a MSCR test at 58 °C, 64 °C, 70 °C and 76 °C, showing that the resistance to permanent deformation of WPU/SBS composite modified asphalt with different WPU content can be characterized by creep recovery rate (R), non-recoverable creep compliance (*J_nr_*) and change rate of *J_nr_* with stress (*J_nr−diff_*).

Multiple stress creep recovery (MSCR) test: 10 creep recovery tests were carried out under shear stress of 0.1 kPa and 3.2 kPa, creeping for 1 s and recovering for 9 s at 58 °C, 64 °C, 70 °C and 76 °C; then, *J_nr_* under different stresses and *J_nr−diff_* were calculated according to the collected strain. The calculation method can be expressed as follows:

(2) $$J_{\mathrm{nr}}=\frac{\varepsilon_{r}-\varepsilon_{0}}{\delta }$$

(3) $$J_{\mathrm{nr}-diff}=\frac{J_{nr(3.2\;\mathrm{kPa})}-J_{nr(0.1\;\mathrm{kPa})}}{J_{nr(3.2\;\mathrm{kPa})}}$$

where,$\;\varepsilon_{0}$ is the initial deformation and $\varepsilon_{r}$ is the residual deformation after recovering for 9 s; *δ* is the shear stress; $J{\;}_{nr}$ is the non-recoverable creep compliance; and $J_{nr-diff}$ is the rate of change of non-recoverable creep compliance with stress.

(3) Bending Beam Rheometer (BBR) Tests

According to ASTM-08 [19], low-temperature performance of WPU modified asphalt was measured with a TE-BBR SD purchased from Cannon Instrument Company (State College, PA, USA) at −12 °C, −18 °C and −24 °C, respectively. Therefore, stiffness modulus (s) and creep rate (m) of samples were measured at low temperature, as well as performance grade (PG) of WPU/SBS composite modified asphalt through rheological property experiment of WPU/SBS composite modified asphalt.

#### 2.4.3. Storage Stability Tests

According to the experimental specification JTJF40, upper and lower layers of WPU/SBS composite modified asphalt were separated by a segregation experiment, and anti-rutting factor G^*^/sin *δ* and segregation index (SI) of upper and lower samples were also obtained by temperature scanning with DSR experimental instrument according to the requirements of ASTMD 7175. Segregation index was taken as the ratio of rutting resistance factors of upper and lower samples separated from WPU/SBS composite modified asphalt.

(4) $$SI={\left[G^{*}/\sin\delta \right]}_{bottom}/{\left[G^{*}/\sin\delta \right]}_{top}$$

In the formula, [G^*^/*sin* δ]*_bottom_* and [G^*^/*sin* δ]*_top_* are rutting resistance factors for upper and lower layers of WPU/SBS composite modified asphalt segregation, respectively [20]. If WPU/SBS composite modified asphalt system is stable, SI value is 1. The higher the segregated SI deviated from 1, the worse the stability and compatibility of WPU/SBS composite modified asphalt.

#### 2.4.4. Microstructure Analysis

(1) Fluorescence Microscope (FM) tests

Fluorescence Microscope (FM) (Opal, Shanghai, China) is the most valuable method to characterize WPU dispersion in WPU/SBS composite modified asphalt [21]. In the experiment, a glass rod was dipped into a high-temperature modified asphalt sample, which was placed in the center of sliding glass, covered with glass on the sliding glass, and put in the oven at 120 °C for 40 min to form a semi-transparent film. Finally, prepared samples were cooled at room temperature, to observe the dispersion of WPU in asphalt under fluorescence microscope.

(2) Fourier transform infrared spectroscopy (FTIR) tests

Attenuated total reflection spectra (ATR) technology was used in Fourier transform infrared spectroscopy (FTIR) (Fine division, Shanghai, China) analysis. FTIR can characterize the chemical structure of polymer modified asphalt, to analyze the PU content [22,23]. In order to explore the changes of functional groups and chemical components of WPU, WPU/SBS composite modified asphalt was scanned with infrared spectroscopy. Whether WPU reacts with asphalt was characterized by measuring the changes of functional groups of WPU/SBS composite modified asphalt, with the wave number of FTIR ranging from 500 cm^−1^ to 4000 cm^−1^ [24].

## 3. Results and Discussion

### 3.1. Basic Performance

#### 3.1.1. Basic Performance of Asphalts

**Figure 2:**
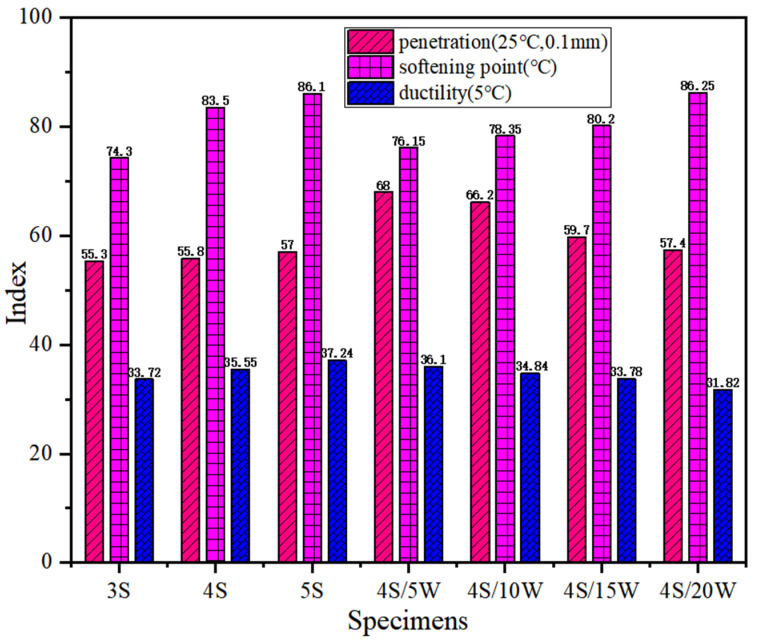
Conventional properties of modified asphalts.

**Figure 3:**
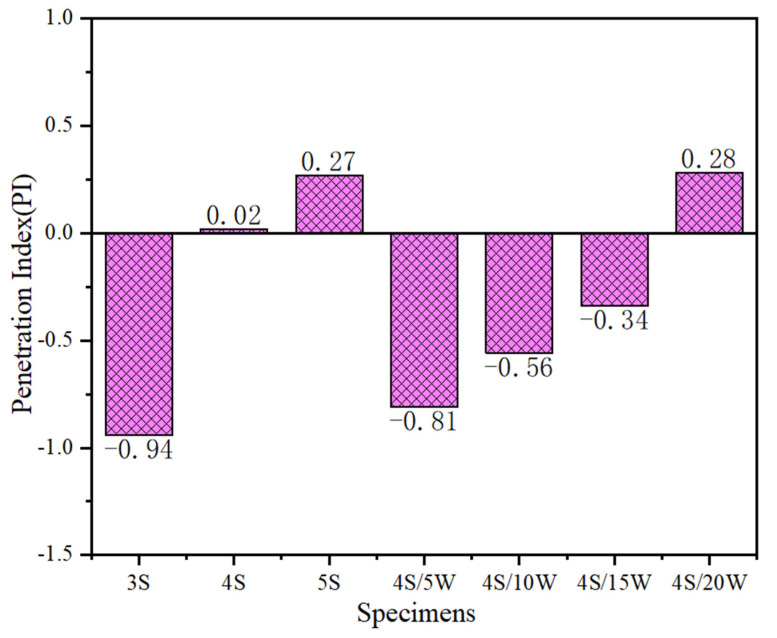
PI of Modified Asphalt.

In the study, the performance of WPU/SBS composite modified asphalt was evaluated accurately with the results of a traditional performance test as control indexes. The effects of SBS and WPU contents on conventional properties of composite modified asphalts are shown in Figure 2 and Figure 3.

The conventional properties of WPU/SBS composite modified asphalt are shown in Figure 2. With the increase of SBS modifier content, the penetration, softening point and ductility at 5 °C of SBS modified asphalt gradually increased, indicating that the addition of SBS modifier contributes to improving the high- and low-temperature performance of SBS modified asphalt. For WPU/SBS composite modified asphalt, penetration and ductility gradually decreased with the increase of WPU content, while the softening point gradually increased, indicating that WPU can harden WPU/SBS composite modified asphalt and improve the high-temperature deformation resistance of asphalt. As the softening points increased, the high-temperature performance of WPU/SBS composite modified asphalt was significantly improved by the incorporation of WPU. The decrease of ductility at 5 °C indicates that the incorporation of WPU may reduce the low-temperature deformation resistance of WPU/SBS composite modified asphalt. Compared with 4% SBS modified asphalt, the penetration was improved in WPU/SBS composite modified asphalt, and the softening point and ductility at 5 °C were reduced as well, indicating that adding WPU can make WPU/SBS composite modified asphalt harden, which is unsuitable for flowing.

As shown in Figure 3, the PI index of SBS modified asphalt gradually increased with the increase of SBS modifier content. It also clearly increased in WPU/SBS composite modified asphalt with the increase of WPU content, suggesting that SBS modifier and WPU can improve the temperature sensitivity of base asphalt. However, compared with 4% SBS modified asphalt, WPU obviously reduced the temperature sensitivity of WPU/SBS modified asphalt, and WPU/SBS composite modified asphalt has sol-gel structure [2] and elastic recovery ability, which is easily affected by temperature.

#### 3.1.2. Results of Brookfield Rotational Viscosity Tests

The viscosity-temperature curve of modified asphalt can characterize the rheological properties of binder, thus reflecting the mixing temperature and compaction temperature of asphalt mixture [10]. In this study, the viscosity of WPU/SBS composite modified asphalt was measured from 135 °C to 175 °C by rotating viscometer.

**Figure 4:**
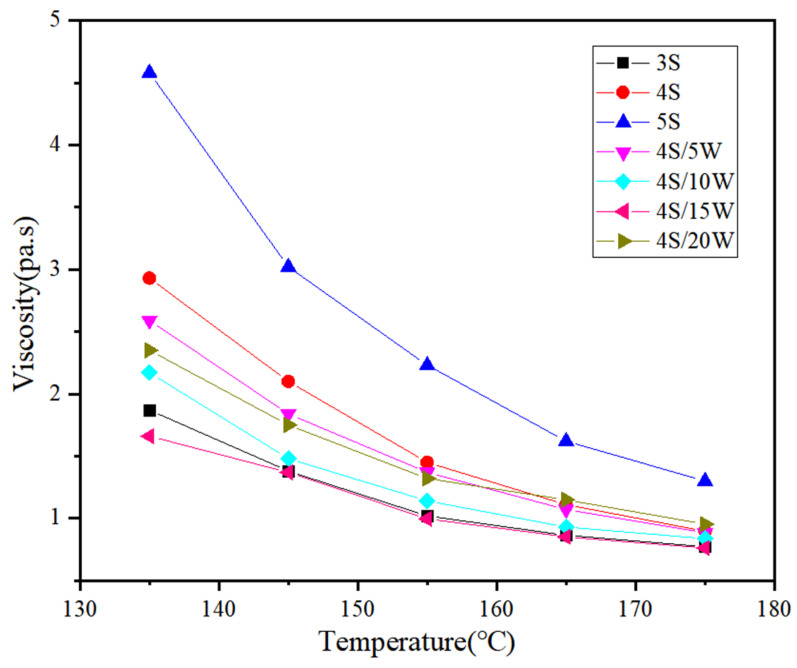
Viscosity of different composite modified asphalts.

As shown in Figure 4, the viscosity of SBS modified asphalt increased with the increase of SBS modifier content. As WPU content increased, the viscosity of WPU/SBS composite modified asphalt decreased first, and then increased; this increase was reduced by WPU when adding a small amount of wet powder, and obviously improved when WPU content was more than 15%. The experimental results showed that with the incorporation of WPU in WPU/SBS composite modified asphalt, WPU destroyed the interweaving between asphalt molecular segments, and made some asphalt molecules free, thus reducing the viscosity of modified asphalt. When WPU content was more than 15%, there was a new interweaving effect between WPU and asphalt molecular segments, with the viscosity of WPU/SBS composite modified asphalt increasing. This was because the viscosity of waste polyurethane was low, and the viscosity of WP/SBS composite modified asphalt was reduced by increasing the content of waste polyurethane. When the content of waste polyurethane was large, the original cross-linking effect was formed between the molecules of WP/SBS composite modified asphalt, and the viscosity increases. Under external load, WPU in WPU/SBS composite modified asphalt has a strong resistance to flow deformation to a certain extent. According to JTGF40-2004 and SHRP asphalt cement standard specifications, the polymer modified asphalt will not exceed 3 Pa at 135 °C [25]. When SBS modifier content was 5%, the rotational viscosity of SBS modified asphalt was 4.58 Pa·s at 135 °C, which did not meet the requirements. When the content of SBS was 4%, the viscosity and performance of SBS modified asphalt were suitable to be WP/SBS composite modified asphalt base material. In the preparation of WPU/SBS composite modified asphalt, 4% SBS modified asphalt was selected as the basis, with different amounts of WPU, to reduce environmental pollution with WPU in asphalt roads and determine the feasibility of replacing some SBS modifiers with WPU as modifier.

### 3.2. Rheological Properties

#### 3.2.1. Temperature Sweep Test

**Figure 5:**
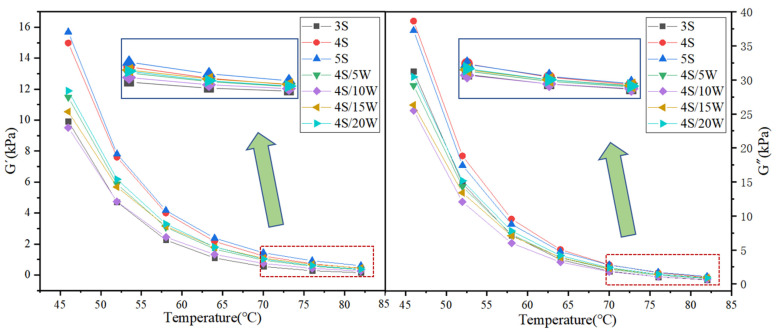
G’ and G” of WPU/SBS composite modified asphalt.

**Figure 6:**
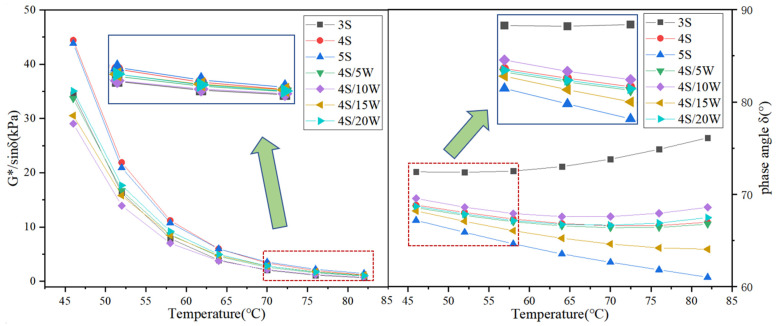
G^*^/sin *δ* and *δ* of WPU/SBS composite modified asphalt.

The rheological parameters, such as storage modulus G’, loss modulus G”, rust resistance factor G^*^/sin *δ* and phase angle *δ*, were obtained by temperature scanning experiments of WPU/SBS composite modified asphalt with DSR [26], which were used to characterize the viscoelasticity and rutting resistance of asphalt binder [27]. The variation of rheological parameters of modified asphalt is presented in Figure 5 and Figure 6.

As shown in Figure 5, with the increase of SBS modifier content, G’ and G″ of SBS modified asphalt increased significantly, with the loss modulus G″ of SBS modified asphalt higher than the storage modulus G’, revealing that the viscoelasticity of SBS modified asphalt can increase with the addition of SBS modifier, especially the viscosity improvement. With the increase of WPU content, G’ and G″ of WPU/SBS composite modified asphalt decreased first, then increased, and decreased again with the increase of test temperature, indicating that adding a small amount of WPU can reduce the viscoelasticity of modified asphalt, because it will destroy the crosslinking effects of SBS modified asphalt. When WPU content was more than 10%, the viscoelasticity of modified asphalt was improved, so that WPU can form a new crosslinking effect in asphalt, thus increasing the viscoelasticity of modified asphalt.

At lower WPU dosage, the asphalt in the modified asphalt was a continuous phase, WPU was a dispersed phase, and WPU was dispersed as a small molecule in the asphalt, which led to a relative increase in the content of small molecules in the modified asphalt. The addition of WPU increases the movement resistance of the modified asphalt, which reduces the high temperature stability of the modified asphalt. With the increase of WPU dosage, the phase state of WPU and asphalt in modified asphalt altered, the small molecules of WPU agglomerated, the micro-phase structure of WPU was brought into full play, and WPU intertwined and cross-linked to form an interlocking network structure, so that the rheological properties of WPU/SBS composite modified asphalt were improved.

As shown in Figure 6, with the increase of SBS content, the anti-rutting factor G^*^/sin *δ* of SBS modified asphalt increased, while the phase angle *δ* decreased [6], which decreased first and then increased in WPU/SBS composite modified asphalt with the increase of WPU content, and the phase angle *δ* first changed on the opposite. Meanwhile, with the increase of test temperature, G^*^/sin *δ* decreased gradually and the phase angle *δ* increased gradually. It was found that with the increase of SBS modifier content, the rutting resistance of SBS modified asphalt can be significantly improved. Also, for WPU/SBS composite modified asphalt, adding a small amount of WPU can change the crosslinking state of composite modified asphalt and reduce the rutting resistance of composite modified asphalt. Similar results were reported by Bazmara et al. [10]. When WPU content was higher than 10%, WPU was uniformly distributed in asphalt, forming a new crosslinking effect and improving the rutting resistance of asphalt. When WPU content in WPU/SBS composite modified asphalt was higher than 10%, the elasticity of composite modified asphalt increased, which is consistent with the analysis of loss modulus G″ and storage modulus G’.

#### 3.2.2. Multiple Stress Creep Recovery (MSCR) Test

**Figure 7:**
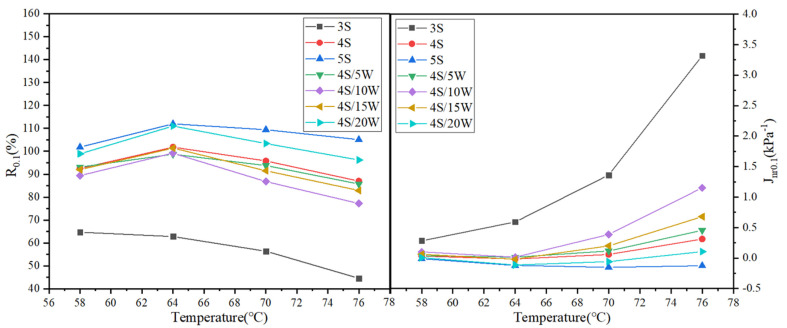
R_0.1_ and *J*_*nr*0.1_ of WPU/SBS composite modified asphalt.

**Figure 8:**
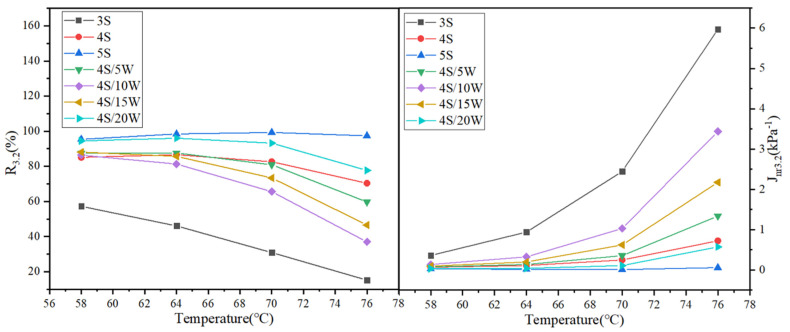
R_3.2_ and *J*_*nr*3.2_ of WPU/SBS composite modified asphalt.

Figure 7 and Figure 8 show the test results of R value and *J_nr_* value of WPU/SBS composite modified asphalt with shear stress of 0.1 kPa and 3.2 kPa, respectively. When the shear stresses were 0.1 kPa and 3.2 kPa, the creep recovery rate R of SBS modified asphalt gradually increased, and the non-recoverable creep compliance *J_nr_* gradually decreased [28], with the increase of SBS modified content. For WPU/SBS composite modified asphalt, R first decreased and then increased with the increase of WPU content, while *J_nr_* first increased and then decreased. Also, the R of modified asphalt decreased gradually, and *J_nr_* increased gradually with the increase of test temperature. The results above showed the variation of *J_nr_* of SBS modified asphalt, which indicates that viscous deformation will decrease with the increase of SBS modifier, namely, the higher the modifier content, the better the resistance to high-temperature deformation. With the increase of WPU content, *J_nr_* of WPU/SBS composite modified asphalt first increased and then decreased, indicating that high-temperature deformation resistance of WPU/SBS composite modified asphalt can be weakened with WPU content less than 10%, and the resistance to permanent deformation of WPU/SBS composite modified asphalt can be obviously improved with WPU content higher than 15%. In addition, this was consistent with asphalt test results [11].

**Figure 9:**
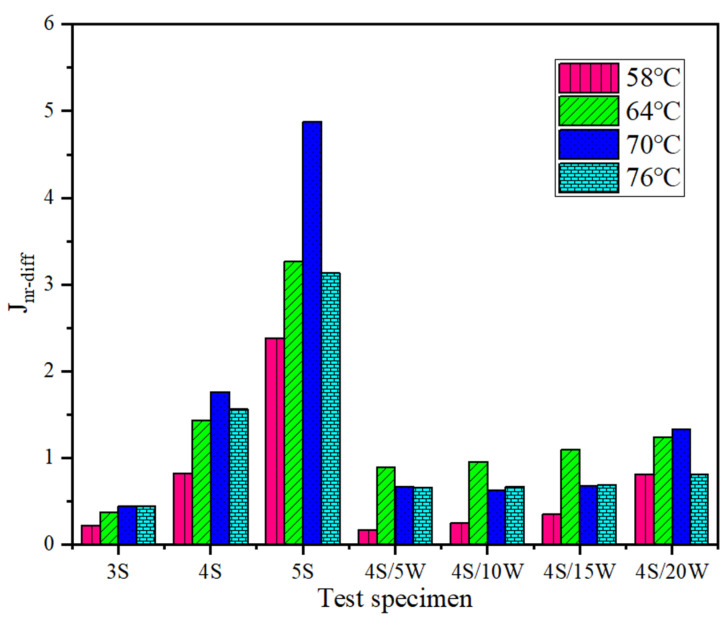
*J_nr−diff_* of WPU/SBS composite modified asphalt.

Figure 9 shows the change rules of *J_nr−diff_* value of WPU/SBS composite modified asphalt. *J_nr−diff_* value of SBS modified asphalt increased with the increase of SBS modifier content, indicating that SBS modified asphalt has better resistance to high-temperature deformation as the content increases, but its viscous deformation is more dependent on shear stress. At different test temperatures, *J_nr−diff_* value of WPU/SBS composite modified asphalt did not change significantly as WPU content increased, indicating that the viscosity deformation of WPU/SBS composite modified asphalt is less dependent on shear stress, because WPU will elastically deform when filling in asphalt, as a buffer when the stress changes. Compared with SBS modified asphalt, *J_nr_* value was lower in WPU/SBS composite modified asphalt, with increasing *J_nr−diff_* value as WPU content increases, which shows that WPU/SBS composite modified asphalt has better high-temperature deformation resistance and lower dependence on shear stress, which is more suitable for the construction of pavements under high temperature, heavy load or long steep slope.

#### 3.2.3. Bending Beam Rheometer (BBR) Test

**Figure 10:**
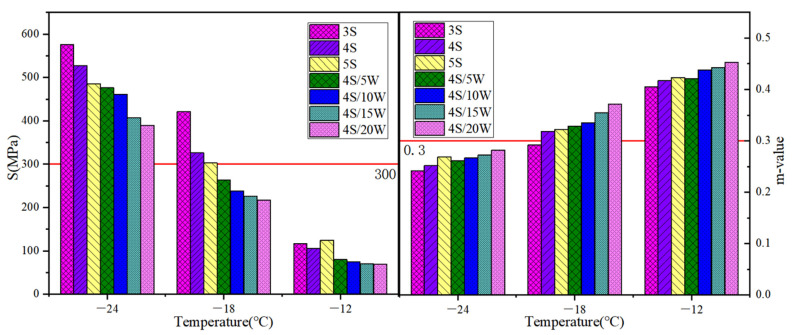
Stiffness and m-value of WPU/SBS composite modified asphalt.

The relationship between temperature and creep stiffness (S) and creep rate (m) of WPU/SBS composite modified asphalt is shown in Figure 10. At −12 °C, −18 °C and −24 °C, creep stiffness (S) and creep velocity (m) of WPU/SBS composite modified asphalt decreased and increased gradually with the increase of temperature [29]. Compared with SBS modified asphalt, S value was obviously smaller in WPU/SBS modified asphalt, with larger m value, which suggests that creep stiffness of modified asphalt at low temperature can be reduced by adding WPU, and the flexibility and creep velocity of modified asphalt increases; thus, WPU/SBS composites can be improved, because WPU can absorb and decompose the external stress of WPU/SBS composite modified asphalt, so that the deformation resistance of modified asphalt can increase and the low-temperature effect can be enhanced [30].

#### 3.2.4. PG

According to DSR and BBR, PG classification of WPU/SBS composite modified asphalt was conducted according to the requirements of G^*^/sin *δ* ≥ 1 kPa, S ≤ 300 MPa and m ≥ 0.3 in American SHRP specifications [29]. As shown in Table 5, the best includes the high-temperature grades of 4 S, 5 S, 4 S/15 W and 4 S/20 W, and the low-temperature grades of 4 S/5 W, 4 S/10 W, 4 S/15 W and 4 S/20 W. Therefore, considering the reuse of WPU, 4 S/15 W is recommended as the suitable WPU/SBS composite modified asphalt in engineering application combined with the conventional indexes and the test results of high- and low-temperature rheological properties.

To sum up, SBS modifier plays a leading role in the modification system and adding WPU can improve the low-temperature performance of modified asphalt, while a small amount of WPU also weakens its high-temperature sensitivity. In order to continue to explore the storage process, WPU/SBS composite modified asphalt was tested and analyzed with DSR equipment.

### 3.3. Results of Storage Stability

WPU, SBS modifier and base asphalt have great differences in chemical composition, morphology and intermolecular force. Therefore, their compatibility is characterized by their storage stability.

**Figure 11:**
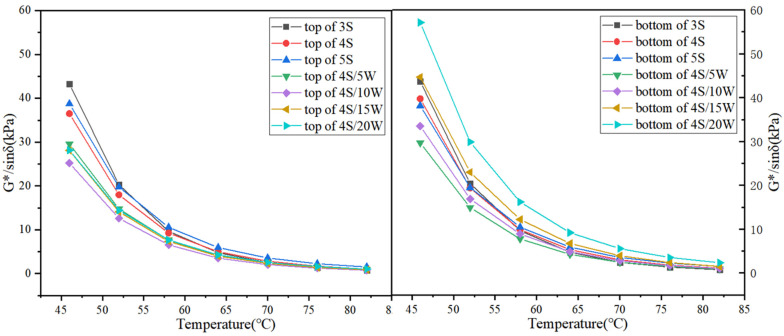
Segregation of G^*^/sin *δ* at top and bottom samples of WPU/SBS composite modified asphalt.

As shown in Figure 11, the rutting resistance factor G^*^/sin *δ* of WPU/SBS composite modified asphalt segregation was significantly larger in the bottom sample than that in the top sample, but changed to a lesser extent in top and bottom samples of SBS modified asphalt segregation as SBS modifier content increased, which increased significantly in the top and bottom samples of WPU/SBS composite modified asphalt segregation as WPU content increased. It was found that adding WPU greatly influences the stability of WPU/SBS composite modified asphalt system, due to the long-term stability of WPU after evenly dispersing in modified asphalt system. In the segregation, part of WPU was separated from asphalt, leading to great differences in rutting resistance factor G^*^/sin *δ* between upper and lower samples. For SBS modified asphalt system, the content of SBS modifier had little effect on the stability of WPU/SBS composite modified asphalt system, which was greatly influenced by WPU content. The more WPU that was added, the more unstable WPU/SBS composite modified asphalt system was, indicating the poor compatibility between WPU and asphalt [31].

**Figure 12:**
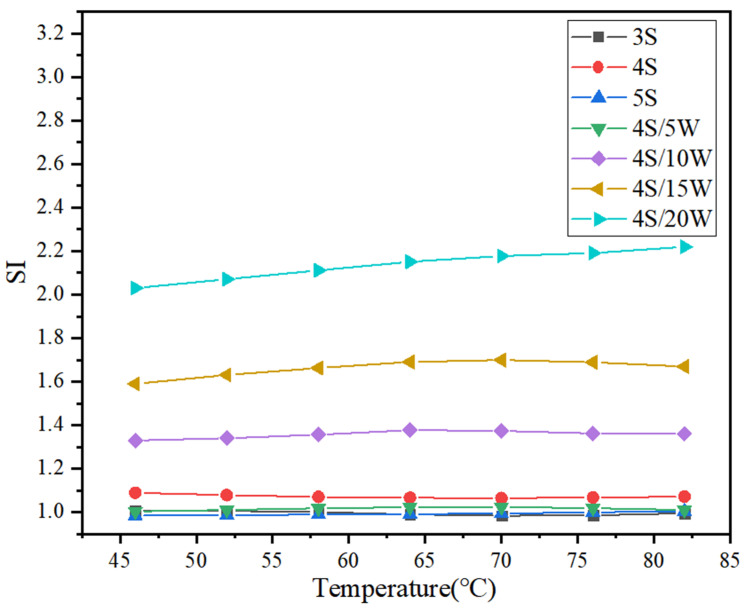
SI of WPU/SBS composite modified asphalt.

Figure 12 shows the change rules of SI value in WPU/SBS composite modified asphalt, revealing that SI value of SBS modified asphalt was close to 1, meaning that SBS modified asphalt system is stable, with little influence from the content of SBS modifier on the stability of modified asphalt system. SI value of WPU/SBS composite modified asphalt deviated from 1 as WPU content increased, indicating the reduced storage stability of WPU/SBS composite modified asphalt when adding WPU, which is consistent with the anti-rutting factor G^*^/sin *δ* of segregation samples in WPU/SBS composite modified asphalt [6,8].

### 3.4. Microstructural Characterization

In order to further explore the microstructure of WPU, FM and FTIR tests were performed on WPU/SBS composite modified asphalt.

#### 3.4.1. Fluorescence Microscope (FM) Test

**Figure 13:**
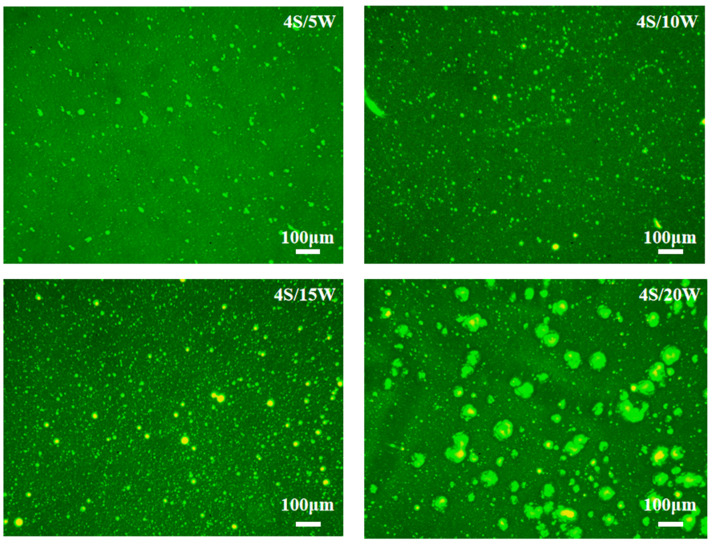
Morphology of WPU/SBS composite modified asphalt.

The dispersion of WPU in WPU/SBS composite modified asphalt is shown in Figure 13. The dots with bright fluorescence were WPU, with asphalt as green background. WPU was a single phase, while asphalt was a dispersed continuous phase. It was seen that WPU particles were uniformly coated by asphalt, and filled in the form of elastic particles, rather than completely dissolved in asphalt [32]. When the content was 5%, 10% and 15%, WPU was uniformly dispersed in asphalt, and the fluorescence spots increased obviously as WPU content increased, while the particle size of WPU increased distinctly when WPU content was 20%, with WPU aggregating in asphalt. Therefore, the viscosity and rheological characteristics of WPU/SBS composite modified asphalt changed obviously. With the increase of WPU content, the colloid content in WPU/SBS composite modified asphalt tended to be saturated, and the unsaturated bonds between WPU formed a crosslinking structure, thus showing the agglomeration phenomenon [21]. Moreover, the strong effects of crosslinking structure can restrict asphalt flow, and improve the external force resistance and low-temperature resistance of asphalt.

#### 3.4.2. Fourier Transform Infrared Spectroscopy (FTIR) Test

**Figure 14:**
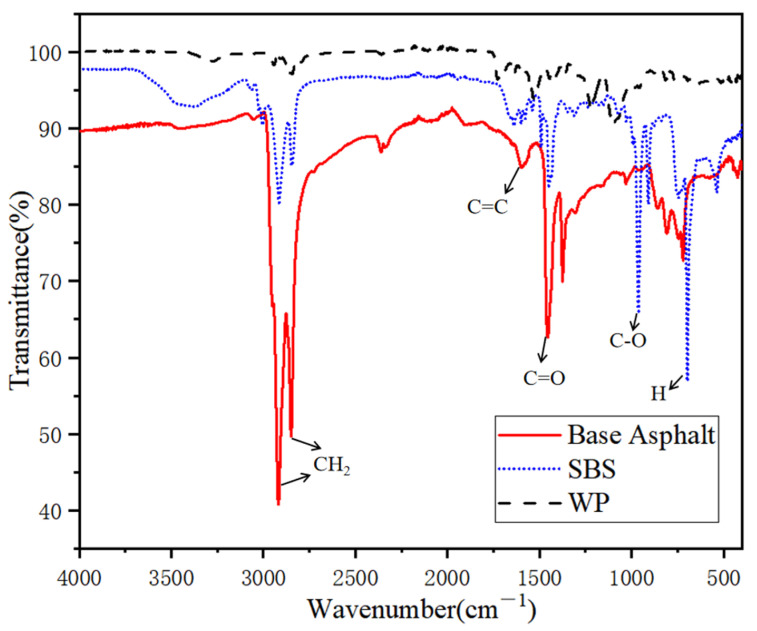
FTIR spectra of SBS and WPU.

**Figure 15:**
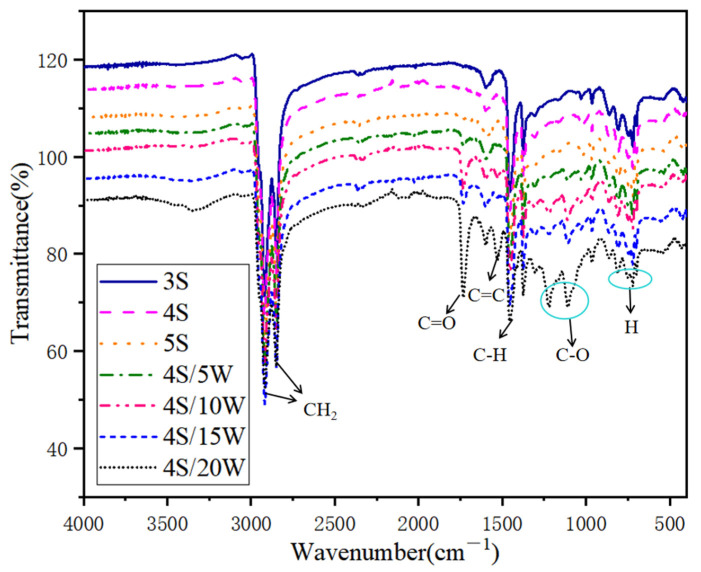
FTIR spectra of WPU/SBS composite modified asphalt.

Whether new chemical bonds can be formed during the preparation of modified asphalt has a significant impact on the properties of modified asphalt [33]. The above experimental results showed that WPU and SBS modifiers improved the performance of modified asphalt, but whether there is a chemical reaction between the two modifiers and base asphalt remains unclear. Therefore, WPU, SBS, matrix asphalt and several WPU/SBS composite modified asphalt were tested by infrared spectroscopy, to characterize the chemical reaction between PU and matrix asphalt, as shown in Figure 14 and Figure 15.

As shown in Figure 14 and Figure 15, the infrared spectrum presented different absorption peaks. The absorption peaks mainly concentrated during 650 cm^−1^~1800 cm^−1^ and 2700 cm^−1^~3000 cm^−1^, which were formed by the swinging vibration of H bond on benzene ring for 650 cm^−1^~900 cm^−1^, the stretching vibration of C-O bond for 1000 cm^−1^~1300 cm^−1^, the vibration of carboxyl group (C-H) in inorganic carbonate for 1453 cm^−1^, the vibration of benzene ring skeleton (C=C) for 1551 cm^−1^,the vibration of carboxyl group (C=O) in inorganic carbonate for 1750 cm^−1^,and the stretching vibration of -CH_2_ group for 2848 cm^−1^ and 2920 cm^−1^. According to the above analysis, WPU mainly consists of carboxyl, unsaturated carbon chain, carbonate, etc. [34,35], which can enhance the aromaticity and polarity of WPU/SBS composite modified asphalt, thus improving the absorption capacity of modified asphalt on aggregate surface and the water damage resistance of WPU/SBS composite modified asphalt mixture.

After comparing the absorption peaks of WPU, SBS, matrix asphalt and WPU/SBS composite modified asphalt, no new chemical bond production of WPU/SBS composite modified asphalt was found, with only physical fusion between WPU and modified asphalt without chemical reaction [36]. Two prominent absorption peaks of WPU were caused by the stretching vibration of C-O bond and benzene ring skeleton (C=C) bond. With the increase of average waste content, the peak height of C-O bond and benzene ring skeleton (C=C) of WPU/SBS composite modified asphalt increased gradually, indicating that the distribution of WPU is uniform, but the content increases without any reaction. When WPU content was 20%, the two peaks in WPU/SBS composite modified asphalt increased significantly, which was mainly caused by the crosslinking and agglomeration of WPU after the excessive content of WPU [28].

## 4. Conclusions

In this study, samples of WPU/SBS composite modified asphalt were successfully prepared by a new preparation process. The rheology, storage stability and microstructure characteristics of WPU/SBS composite modified asphalt at high and low temperature were studied and discussed by routine performance test, DSR test, BBR test and microscopic analysis test. According to the above study, the following conclusions are drawn:

(1) With WPU, the penetration, ductility and PI index of composite modified asphalt decreased, as softening points and viscosity of composite modified asphalt increased, with higher viscosity, but lower temperature sensitivity. The feasibility of using WPU as modifier instead of part of SBS was confirmed by viscosity performance.
(2) The incorporation of a small amount of WPU changed the crosslinking state of composite modified asphalt and reduced its rutting resistance. When WPU content was more than 15%, WPU formed a new crosslinking effect in asphalt, which enhanced the rutting resistance of asphalt and improved low-temperature performance of WPU/SBS composite modified asphalt. According to the high- and low-temperature rheological properties, PG grade of WPU/SBS composite modified asphalt was determined.
(3) Adding WPU significantly improved the resistance to permanent deformation of WPU/SBS composite modified asphalt and reduced the dependence of viscous deformation on shear stress, which was more suitable for the construction of pavements with high temperature, heavy load or long steep.
(4) SI value of upper and lower layers of WPU/SBS composite modified asphalt segregation deviated from 1, indicating that the storage stability of WPU/SBS composite modified asphalt was reduced by WPU content.
(5) Through the study of the microstructure of WPU/SBS composite modified asphalt, WPU was mixed into the modified asphalt and evenly coated by asphalt, without new chemical bonds and chemical reactions. When WPU content was 20%, WPU agglomerated in asphalt, which affected the storage and engineering application of WPU/SBS composite modified asphalt. After comprehensive consideration, 4 S/15 W was determined as the suitable type of WPU/SBS composite modified asphalt.

## Figures and Tables

**Table 1:** Properties of base asphalt.

Parameter	Results	Standard in China (JTG E20-2011)
Penetration (0.1 mm)	68	T0604
Softening point (°C)	47.8	T0606
Ductility (15 °C) (cm)	>100	T0605
Dynamic viscosity at 60 °C (Pa s)	167.5	T0620
Density (15 °C) (g/cm^−3^)	1.047	T0603
Flash point (°C)	345	T0611

**Table 2:** The four components of base asphalt.

Asphalt	Asphaltenes (%)	Resins (%)	Aromatics (%)	Saturates/ (%)	Colloidal Index
SK	11.69	29.97	48.68	9.66	3.68

**Table 3:** Properties of WPU.

Parameter	Results	Standards
Density (g/cm^3^)	1.16	DIN 53479 [13]
Tensile strength (N/mm^2^)	42	DIN 53504-S2 [14]
Tear strength (N/mm^2^)	64	DIN 53515 [15]
Softening point (°C)	71	ISO 306 [16]

**Table 4:** Basic properties of SBS.

Parameter	Results	Standards
Proportion (g/cm^3^)	0.94	ISO 2781
Melt flow index (g/10 min)	6	ASTM D1238
Tensile strength (MPa)	160	ASTM D412
Elongation (%)	680	ASTM D412

**Table 5:** The PG grading of WPU/SBS composite modified asphalt.

Sample Types	PG Grade	G^*^/sin *δ*/kPa	S/MPa	m
3 S	76-22	1.12	117	0.405
4 S	82-22	1.19	106	0.318
5 S	82-22	1.44	124	0.322
4 S/5 W	76-28	1.55	263	0.328
4 S/10 W	76-28	1.24	238	0.335
4 S/15 W	82-28	1.15	226	0.355
4 S/20 W	82-28	1.04	217	0.372

## Data Availability

Not applicable.
